# Retinal Vascular Caliber Is Associated with Cardiovascular Biomarkers of Oxidative Stress and Inflammation: The POLA Study

**DOI:** 10.1371/journal.pone.0071089

**Published:** 2013-07-26

**Authors:** Vincent Daien, Isabelle Carriere, Ryo Kawasaki, Jean-Paul Cristol, Max Villain, Pierre Fesler, Karen Ritchie, Cecile Delcourt

**Affiliations:** 1 Inserm, U1061, Montpellier, France; 2 Université Montpellier 1, U1061, Montpellier, France; 3 Department of Ophthalmology, Gui De Chauliac Hospital, Montpellier, France; 4 Department of Public Health, Yamagata University Faculty of Medicine, Yamagata, Japan; 5 Department of Biochemistry, Lapeyronie Hospital, Montpellier, France; 6 Department of Internal Medicine, Hôpital Lapeyronie, Montpellier, France; 7 Faculty of Medicine, Imperial College, St Mary's Hospital, London, United Kingdom; 8 Inserm, Centre Inserm U897-Epidemiologie-Biostatistique, Bordeaux, France; 9 Université Bordeaux, ISPED, Bordeaux, France; University Medical Center Utrecht, The Netherlands

## Abstract

**Purpose:**

Retinal vascular caliber has been linked with increased cardiovascular risk and is predictive of cardiovascular pathology, including stroke and coronary heart disease. Oxidative stress, as well as inflammatory mechanisms, plays a major role in the pathogenesis and progression of atherosclerosis, plaque rupture and vascular thrombotic propensity. The purpose of this study is to explore the relationship between retinal vascular calibers and biomarkers of oxidative stress and inflammation, in subjects free of cardiovascular pathology.

**Patients and Methods:**

Cross-sectional analysis from a community-dwelling cohort comprising 1224 individuals aged 60 years and over, without a history of coronary or peripheral artery disease or stroke. Retinal vascular caliber was measured from fundus photographs using semi-automated standardized imaging software. Oxidative stress was evaluated using plasma superoxide dismutase 2 and glutathione peroxidase (GPx-3) activities, and inflammatory state was assessed using plasma high sensitivity C-reactive protein (hsCRP) and orosomucoid.

**Results:**

In a multivariate model controlling for cardiovascular risk factors, larger retinal arteriolar caliber was independently related to higher level of GPx-3 activity (p = 0.003) whereas larger venular caliber was associated with higher levels of hsCRP (p = 0.0001) and orosomucoid (p = 0.01).

**Conclusion:**

In the present study, biomarkers of oxidative stress regulation and inflammation were independently associated with retinal vascular calibers. This suggests that an assessment of retinal vessels may offer early and non-invasive detection of subclinical vascular pathology.

## Introduction

Pathologies associated with atherosclerosis, including coronary thrombosis, stroke and peripheral arterial disease, continue to be among the leading causes of death worldwide [1]. Since the underlying pathophysiology develops in most patients long before cardiovascular disease is diagnosed, simple examinations, able to detect the early vascular remodeling process leading to disease, would be of considerable value for early intervention and prevention. Previous studies have suggested that microcirculatory changes are closely linked to cardiovascular modifications in humans [2], [3]. Retinal photography, by allowing a direct observation of retinal vessels, may thus constitute a practical and noninvasive method for the examination of early changes in human microcirculation. Changes in the caliber of retinal vessels have been shown to reflect the cumulative effects of birth weight [4], [5], the aging process [6], cardiovascular risk factors [7], [8], renal function [9], and genetic factors [10], [11].

In meta analyses from epidemiological studies, wider retinal venules and narrower arterioles were associated with an increased risk of coronary heart disease in women [12] and an increased risk of global cardiovascular mortality [13], while wider retinal venular caliber predicted stroke [14].

However the artherosclerosis process begins long before the onset of cardiovascular events and the question remains if retinal microvasculature may be a marker of early modifications.

Inflammatory mechanisms play a major role in the pathogenesis and progression of atherosclerosis, plaque rupture and vascular thrombotic propensity. Indeed, the dynamic inflammation model has supplanted the previously held view of atherosclerosis as a passive deposition of debris in the arterial wall [15]–[17]. Lesion initiation involves the expression of adhesion molecules on the surface of the endothelial cells and the recruitment and directed migration of blood-borne inflammatory cells into the artery wall. Numerous mediators contribute to atherogenesis, including chemokines, cytokines, growth factors, proteases, adhesion molecules, hemostasis regulators, and receptors, and their interactions may regulate plaque progression and instability. Two major markers of inflammation are C-reactive protein (CRP) and orosomucoid which are synthesized by hepatocytes. Their levels rise at the acute-phase of inflammation, rapidly for CRP and slowly for orosomucoid. With effective therapy of the inflammation process (e.g. antibiotics for an infection) the concentration of CRP rapidly decreases to normal values within 24 h, whereas orosomucoid decreases more slowly, over several days [18].

Oxidative stress is also recognized as a key pathogenic process in cardiovascular disease [19], [20]. There is evidence that major cardiovascular risk factors enhance the production of vascular reactive oxygen species which are involved in thrombosis [21] and endothelial barrier dysfunction [22]. There are several enzyme systems that catalyze reactions to neutralize free radicals and reactive oxygen species. These enzymes include catalase, superoxide dismutase (SOD) and glutathione peroxidase (GPX). Catalase is an intracellular antioxidant enzyme that is very effective in high-level oxidative stress and protects cells from hydrogen peroxide. Among the many markers of oxidative stress, we chose to use SOD and GPX enzymes, both of which play a major role in the regulation of the redox state of vascular cells [23] that could potentially impact retinal microcirculation. Recently, low levels of GPx-3 activity has been associated with platelet-dependent thrombosis [21], increased risk for arterial stroke in young adults and children [24], [25] and coronary artery disease [26]–[28].

While both inflammation and oxidative stress biomarkers have been extensively studied in relation to atherosclerosis and cardiovascular disease, few studies have investigated their potential relationship with microcirculation. Systemic inflammatory markers have been significantly associated with larger retinal venules [29]–[31], but results in relation to retinal arteriolar calibers are conflicting [32], [33]. To our knowledge there are no published data on the relationship between antioxidant enzymes and retinal microcirculation.

The aim of the current research is thus to examine the biomarkers of oxidative stress and inflammation in relation to retinal vascular calibers, adjusting for other cardiovascular risk factors. In order to assess the early stages of atherogenesis and vascular remodeling, we focused our analyses on an elderly population with no history of coronary heart disease, peripheral artery disease or stroke.

## Patients and Methods

### Study Population

The present study is a cross-sectional analysis from the Pathologies Oculaires Liées à l'Age (POLA) Study, a prospective study aimed at identifying the risk factors of age-related eye diseases. The study design has been published elsewhere [34]. For inclusion in the study, participants needed to be resident of Sète (South of France) and aged 60 years or over. According to the 1990 population census, there were almost 12000 eligible residents. The population was informed of the study through the local media and the study organizers also contacted 4543 residents individually by mail and telephone, using the electoral roll. The baseline examinations took place in a mobile unit equipped with ophthalmologic devices. Between June 1995 and July 1997, 2584 participants were recruited.

From this sample, 391 (15.1 %) individuals were excluded due to suspicion of atherosclerosis if the subject declared a history of complications of atherosclerosis whether they were related to stroke or coronary and peripheral artery disease. This was assessed with a standardized questionnaire on medical antecedents. From the 2193 persons 120 individuals were excluded due to missing data (10 for interview data, 110 for biochemical data). For 81 persons, the retinal photographs were not performed and for 307 the photographs were not available because of technical failure or opacities. For 461 participants, the photographs were of insufficient quality for retinal vascular caliber measurement with semi-automated standardized software (rejection mainly due to default of centering on the optic disc and inability to identify major vessels). Therefore, all statistical analyses are based on 1224 subjects, among the 2193 (56 %) subjects free of clinical atherosclerosis. This research was approved by the ethics committee of the University Hospital of Montpellier, France and written informed consent was obtained from each participant [35], [36].

### Retinal Photography and Measurement of Retinal Vascular Caliber

After pupil dilation, one 50° color retinal photograph (Kodacolor Gold 100 ASA, Eastman Kodak Company, Rochester, NY) was taken of each eye. After film processing, retinal photographs were converted to digital images by a high resolution scanner (Nikon LS2000, Nikon Inc., Tokyo, Japan) using standard settings for all photographs.

Diameters of all vessels coursing through a specified area (0.5–1 disc diameter surrounding the optic disc) were measured by one of the authors (VD), blinded from other data and using image analysis software (IVAN, Department of Ophthalmology Visual Science, University of Wisconsin, Madison, WI). The calibers of the central retinal artery and vein were estimated using the 'Big-6 formula' and summarized as the central retinal artery and vein equivalents (CRAE and CRVE) [37], [38] representing the average diameter of respectively the arterioles and venules of the eye. The reproducibility of retinal vascular measurements was high, with intragrader correlation coefficients of 0.97 (95% confidence interval [CI]: 0.96–0.98) for CRAE and 0.95 (95% CI: 0.94–0.96) for CRVE. Due to the high inter-eye correlation reported in previous studies [39], we analysed only one eye per subject (the right eye, or, if unavailable or ungradable, the left eye).

### Assessment of biomarkers of inflammation and oxidative stress

Oxidative stress was assessed using two biomarkers: SOD-2 and GPx-3 activity, plasma antioxidant enzymes highlighted as being important in regulating platelet activity, endothelial function, platelet-dependent thrombosis, and vascular disease-associated thrombosis [40], [41]. The measurement of plasma GPx-3 activity was performed using the enzyme-linked immunoassay Bioxytech (Bioxytech pl-GPx-3- EIA, OXIS International SA, Portland, OR). Red blood cell SOD-2 was measured by a spectrophotometric assay (Bioxytech SOD-525; OXIS International SA). Plasma orosomucoid were determined by immunoturbidimetric methods, while hs-CRP concentration was determined by latex enhanced immunoturbidimetric method using reagents from Olympus (Rungis, France) on an Olympus AU2700 biochemistry analyzer [42]. Biologic measurements were made from fasting blood samples taken at the participant's home on the morning of the examination before usual medication intake. Coefficients of variation for measurements of plGPX and SOD were, respectively, 6% and 3% [36].

### Covariates

Blood pressure was measured once, in a seated position, after at least 5 minutes rest. Hypertension was defined as known treated hypertension confirmed by current use of antihypertensive medications and/or a systolic blood pressure (BP) of ≥140 mmHg and/or diastolic blood pressure of ≥90 mmHg. Diabetes was defined as a self-reported history of diabetes confirmed by current antidiabetic therapy and/or fasting blood glucose of ≥7 mmol/L. Body mass index (BMI) was defined as weight/height^2^ in kg/m^2^. A BMI between 25 and 30 kg/m^2^ was classified as overweight, and a BMI greater than 30 kg/m^2^ as obese. Fasting blood samples were obtained for the measurement of serum creatinine and plasma glucose. Plasma triglycerides and total cholesterol levels were measured by routine enzymatic methods with a reagent purchased from Boehringer Ingelheim Laboratories (Ingelheim, Germany). Renal function was assessed from estimates of glomerular filtration rate using Modification of Diet in Renal Disease (MDRD) formula, based on plasma creatinine [43].

### Statistical Analysis

Comparisons between included and excluded participants were performed using Chi-squared and Student's t-test or Wilcoxon test depending on data distribution. Retinal vascular caliber (CRAE and CRVE) was normally distributed in our sample. Analyses of covariance (ANCOVA) and linear regression models were subsequently used to determine the association of cardiovascular factors with retinal vascular calibers adjusted for age and gender. A test for linearity was performed when treating risk factors continuously. The hsCRP displayed a skewed distribution, and normality was obtained after natural logarithmic (log_e_) transformation.

Potential confounders retained in the final multivariate models were covariates significantly associated with either CRAE or CRVE in the initial models. If collinearity between 2 variables was found, only one parameter was included in the model (eg. for inflammation parameters). To compare associations between arteriolar and venular calibers directly, we used the same final multivariable models for both. In these models, we also reported the standardized regression coefficient (standardized β). Standardized β estimates were computed by multiplying the original estimates by the sample standard deviation of the regressor variable and dividing by the sample standard deviation of the dependent variable.[44] in order to compare relative strength of associations.

Because the distribution of GPx-3 significantly departed from normality, age- and sex-adjusted associations of GPx-3 with other cardiovascular risk factors were estimated using logistic regression, and being above the median GPx-3 as the dependent variable, and age, sex and the variable of interest as the independent variables. Odds ratios (ORs) are expressed per unit change defined close to the standard deviation of the parameter. All analyses were performed using Statistical Analysis Software (SAS version 9.2; SAS Institute, Cary, NC).

## Results

Characteristics of the study sample are shown in **Table** **1**. The age (Interquartile Range) of the study sample was 68.6 [64–72] years with 41.3 % males, 11.3 % diabetic and 60.0% hypertensive individuals. In the total cohort, the mean (± standard deviation [SD]) central retinal arteriolar caliber was 141.0 microns (±16.7) and venular caliber 202.8 microns (±21.1). When participants were compared with the subjects who could not be included because of missing data, the only significant difference was age (71.7 [66.2–75.9] years in the excluded subjects versus 69.3 [64.4–72.9] years, p = 0.001 in the participants). No differences were found between included and excluded persons for cardiovascular risk factors, inflammation or oxidative stress parameters. **Table** **2** shows the associations of individual cardiovascular risk factors with retinal vascular calibers, after adjustment for age and gender. Smaller retinal arteriolar caliber was related to older age, male gender, hypertensive status and poorer renal function. Current smokers had larger retinal arteriolar caliber. Smaller retinal venular caliber was associated with older age and poorer renal function. Larger retinal venular caliber was related to greater BMI, current cigarette smoking and lower HDL.

**Table 1:** Characteristics of the participants in the POLA Study.

Characteristics of the Participants in the POLA Study	N = 1224 Included	N = 969 Not included*	P value
**Age, y, median (in terquartile range)**	69.3 [64.4–72.9]	71.7 [66.2–75.9]	0.001
**Male (% )**	41.3	41.9	0.63
**Diabetes (% )**	11.3	9.1	0.10
**Hypertension (% )**	60.0	62.0	0.24
**Smoking**			
Never (% )	60.3	60.2%	0.33
Current (% )	9.7	11.2%	
Past (% )	30.0	28.7%	
**Body mass index, kg/m^2^, mean (SD)**	26.5 (4.1)	26.1 (4.1)	0.08
**Fasting blood glucose (mmol/l), mean (SD)**	5.75 (1.36)	5.75 (1.45)	0.21
**Total cholesterol (mmol/l), mean (SD)**	5.80 (1.12)	5.74 (1.03)	0.39
**HDL cholesterol (mmol/l), mean (SD)**	1.40 (0.38)	1.38 (0.37)	0.21
**Triglycerides (mmol/l), mean (SD)**	1.26 (0.74)	1.28 (1.04)	0.89
**eGFR (ml/min/1.73m^2^), mean (SD)**	72.8 (18.0)	75.3 (39.2)	0.15
**HsCRP (mg/l), mean (SD)**	3.62 (0.33)	3.64 (0.41)	0.93
**Orosomucoid (g/l), mean (SD)**	0.82 (0.21)	0.81 (0.22)	0.16
**SOD- 2, mean (SD)**	1.21 (0.31)	1.24 (0.32)	0.18
**GPx-3Activity, mean (SD)**	678.9 (233.1)	665.1 (213.0)	0.19
**Central retinal arteriolar caliber (micron), mean (SD)**	141.0 (16.7)	–	–
**Central retinal venular caliber (micron), mean (SD)**	202.8 (21.1)	–	–

SD: standard deviation; HDL: high-density-lipoprotein; eGFR: estimated Glomerular Filtration Rate from MDRD (Modification of the Diet in Renal Disease) formula; HsCRP: high-sensitivity C-reactive protein.

* 120 individuals were excluded due to missing data (10 for interview data, 110 for biochemical data). For 81 persons, photographs were not performed and for 307 the photographs were not available because of technical failure or opacities. For 461 participants, photographs were of insufficient quality for retinal vascular caliber measurement with semi-automated standardized software (rejection mainly due to default of centering on the optic disc).

**Table 2:** Relationship of retinal vascular caliber with cardiovascular risk factors adjusted for age and gender.

	N	Retinal Arteriolar Caliber*			Retinal Venular Caliber*		
Cardiovascular Risk Factors	1224	Mean	Standard Error	P ANCOVA†	Mean	Standard Error	P ANCOVA†
**Age**							
60–64	351	142.75	0.85	<.0001	206.22	1.12	<.0001
65–69	380	141.31	0.82		203.84	1.07	
70–74	279	138.35	0.96		201.51	1.26	
75–90	214	136.49	1.10		197.97	1.44	
**Gender**							
Men	505	135.57	0.72	<.0001	202.84	0.94	0.45
Women	719	143.88	0.60		201.94	0.79	
**Diabetes**							
Absent	1086	139.92	0.51	0.55	202.33	0.67	0.87
Present, Duration of <10 years	86	138.20	1.73		202.14	2.27	
Present, Duration of ≥10 years	52	138.60	2.22		203.83	2.91	
**Cigarette smoking**							
Never	738	139.90	0.69	0.0008	200.81	0.91	0.006
Current	119	144.47	1.49		207.73	1.96	
Past	367	138.07	0.89		203.35	1.17	
**Body mass index (kg /m^2^)**							
Normal	445	140.27	0.78	0.56	200.83	1.02	0.0005
Overweight	577	139.64	0.67		201.76	0.88	
Obese	202	138.84	1.13		207.53	1.47	
**Hypertension status**							
Normotensive	489	141.93	0.76	0.0002	203.61	0.99	0.12
Hypertensive	735	138.41	0.59		201.66	0.78	
**Total cholesterol (mmol/l)**							
1st Quartile, <5.05	312	140.74	0.91	0.53	203.87	1.19	0.44
2nd Quartile, 5.05–5.75	303	138.97	0.92		201.14	1.21	
3rd Quartile, 5.76–6.48	306	139.26	0.93		202.07	1.22	
4th Quartile, >6.48	303	139.87	0.94		202.37	1.23	
**HDL cholesterol (mmol/l)**							
1st Quartile, <1.13	293	139.51	0.96	0.58	204.69	1.26	0.09
2nd Quartile, 1.13–1.35	340	140.73	0.87		202.89	1.14	
3rd Quartile, 1.36–1.61	292	139.39	0.96		200.95	1.26	
4th Quartile, >1.61	299	139.07	0.98		200.54	1.28	
**Triglycerides (mmol/l)**							
1st Quartile, <0.80	322	140.76	0.90	0.28	202.49	1.19	0.26
2nd Quartile, 0.80–1.07	302	138.85	0.94		200.44	1.23	
3rd Quartile, 1.08–1.52	299	140.40	0.92		202.75	1.21	
4th Quartile, >1.52	301	138.81	0.93		203.77	1.22	
**eGFR** ** **(ml/min/1.73m^2^)**							
1st Quartile, <62.7	305	138.14	0.95	0.007	200.85	1.23	0.005
2nd Quartile, 62.6–70.3	305	139.10	0.93		201.24	1.25	
3rd Quartile, 70.4–78.6	307	140.48	0.94		202.75	1.22	
4th Quartile, >78.6	307	141.21	0.93		204.58	1.22	

* **Retinal vascular calibers in micron, †ANCOVA: analysis of covariance.**

** **eGFR**, estimated Glomerular Filtration Rate from MDRD (Modification of the Diet in Renal Disease) formula.

The independent associations of inflammation and oxidative stress parameters with retinal vascular calibers are shown in **Table** **3**, adjusting for age and gender in **model** **1**, and for all potential confounding factors (current smoking, body mass index, hypertension, HDL cholesterol and estimated glomerular filtration rate) and high-sensitivity C-Reactive Protein (hsCRP) for SOD-2 and GPX-3 activity, and GPX-3 activity for hsCRP and orosomucoid in **model** **2**.

**Table 3:** Associations of retinal vascular calibers with biochemical parameters.

			Retinal Arteriolar Caliber (microns)			Retinal Venular Caliber (microns)		
	Risk Factors	Unit Change	β ± SE	Sβ	Pr > |t|	β ± SE	Sβ	Pr > |t|
**Model 1** *	**log_e_CRP**	Per 0.1 log	0.14±0.09	0.04	0.14	0.58±0.12	0.13	0.0001
	**Orosomucoid**	Per 0.2 g/l	0.11±0.4	0.008	0.78	1.28±0.5	0.06	0.02
	**SOD- 2**	Per 0.3 UM	−0.86±0.5	−0.05	0.07	0.84±0.6	0.04	0.16
	**GPx-3 Activity**	Per 200 UM	1.33±0.4	0.09	0.0008	0.74±0.5	0.04	0.15
**Model 2** †	**log_e_CRP**	Per 0.1 log	0.18±0.1	0.05	0.07	0.51±0.1	0.12	0.0001
	**Orosomucoid**	Per 0.2 g/l	0.10±0.4	0.007	0.85	1.26±0.4	0.06	0.01
	**SOD- 2**	Per 0.3 UM	−0.74±0.4	−0.04	0.09	0.70±0.7	0.05	0.22
	**GPx-3 Activity**	Per 200 UM	1.20±0.4	0.08	0.003	0.61±0.5	0.03	0.23
**Model 3** §	**log_e_CRP**	Per 0.1 log	0.17±0.2	0.05	0.08	0.54±0.1	0.12	0.0001
	**Orosomucoid**	Per 0.2 g/l	0.10±0.4	0.007	0.85	1.34±0.6	0.07	0.01
	**SOD- 2**	Per 0.3 UM	−0.63±0.4	−0.03	0.12	0.59±0.8	0.04	0.35
	**GPx-3 Activity**	Per 200 UM	1.08±0.4	0.08	0.01	0.79±0.6	0.04	0.15

**Abbreviations**: **logeCRP**, logarithmic of high-sensitivity C-Reactive Protein; **SOD- 2**, superoxide dismutase 2; **GPx-3**, glutathione peroxidase activity; **β**, regression coefficient estimated by multivariate linear regression, **SE β**; standard error β; **Sβ**, standardized regression coefficient (computed by multiplying the original estimates by the sample standard deviation of the regressor and divising by the sample standard deviation of the retinal vascular caliber).

* **Model 1:** gender-adjusted association of retinal vascular caliber.

† **Model 2:** Multivariate association of retinal vascular caliber adjusted for age (years), gender (male vs. female), hypertension status (hypertensive vs. normotensive), current smoking status (current vs. never/past), body mass index (kg/m2), high-density-lipoprotein cholesterol (mmol/l), estimated glomerular filtration rate (Modification of the Diet in Renal Disease, ml/min/1.73 m2), and logarithmic of high-sensitivity C-Reactive Protein for SOD-2 and GPX-3, and GPX-3 for logeCRP and Orosomucoid.

§ **Model 3 :** model 2 after exclusion of 138 diabetic subjects.

**Figure 1:**
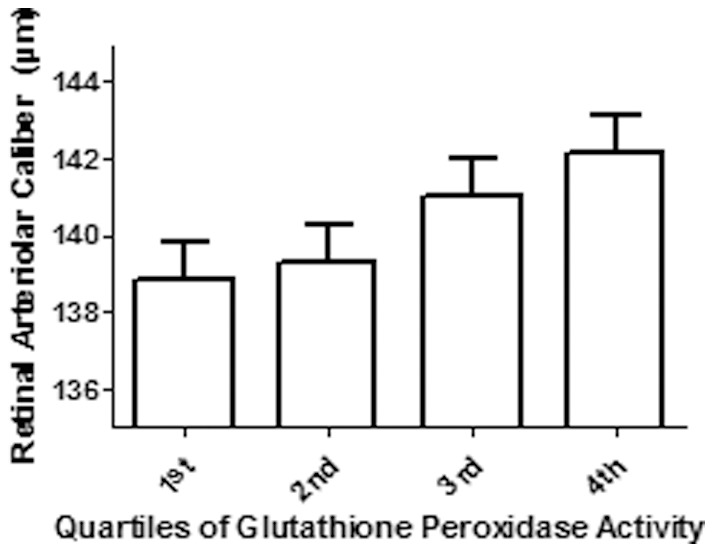
Mean + standard error retinal arteriolar caliber according to quartiles of plasma glutathione peroxidase activity, adjusted for age (years), gender (male vs. female), hypertension status (hypertensive vs. normotensive), current smoking status (current vs. never/past), body mass index (kg/m^2^), high-density-lipoprotein cholesterol (mmol/l), estimated glomerular filtration rate (Modification of the Diet in Renal Disease, ml/min/1.73m^2^), logarithmic of high-sensitivity C-Reactive Protein. P-value for the analysis of covariance (ANCOVA) = 0.003.

After adjustment for age and gender (**model** **1**), retinal arteriolar caliber increased with increasing GPx-3 activity, but was not significantly associated with SOD2, hsCRP or orosomucoid. The results were not affected by further adjustment by potential confounders (**model** **2**). As shown in **Figure 1**, higher quartiles of plasma GPx-3 activity was found to be associated with larger retinal arteriolar caliber (p = 0.003), even after adjustment for potential confounding factors.

After adjustment for age and gender (**model** **1**), retinal venular caliber increased with increasing hsCRP and orosomucoid. Both associations remained statistically significant after further adjustment for potential confounders (**model** **2**). We detected no significant interactions of hsCRP, orosomucoid or GPx-3 activity with other parameters of multivariate analysis.

Since diabetes mellitus is a major driver of oxidative stress, we performed model 3 for which 138 patients with diabetes mellitus were excluded **(** **Table** **3** **)**. This did not significantly affect the associations between retinal vascular caliber and cardiovascular biomarkers of oxidative stress or of inflammation.

Logistic regression was used to model the associations between risk factors and the odds of being above the median for GPX-3 activity **(** **Table** **4** **web only file)**. A higher GPx-3 activity was associated with younger age (OR [odds ratio]  = 0.85; 95% CI [confidence interval]  = 0.77, 0.94), normotensive status (OR = 0.71; 95% CI = 0.56, 0.90), lower BMI (OR = 0.77; 95% CI = 0.67, 0.89), higher HDL-cholesterol (OR = 1.25; 95% CI = 1.10, 1.42), lower triglycerides (OR = 0.93; 95% CI = 0.88, 0.98) and greater renal function (OR = 1.16; 95% CI = 1.05, 1.29).

**Table 4:** Age and gender-adjusted associations of glutathione peroxidase activity with cardiovascular risk factors (web only).

Risk Factors	Unit Change	Age and gender-adjusted OR (95% CI)*	P
**Age**	Per 5 y	0.85 (0.77–0.94)	0.0008
**Gender**	Male vs. female	1.01 (0.80–1.27)	0.91
**Hypertension**	HT vs. NT	0.71 (0.56–0.90)	0.005
**Diabetes**	Absent vs. present	1.10 (0.69–1.74)	0.67
**Cigarette smoking**	Current vs.never/past	0.93 (0.63–1.38)	0.75
**Body Mass Index**	Per 5 kg/m^2^	0.77 (0.67–0.89)	0.0004
**Total cholesterol**	Per 0.8 mmol/l	0.98 (0.90–1.06)	0.64
**HDL cholesterol**	Per 0.4 mmol/l	1.25 (1.10–1.42)	0.0007
**Triglycerides**	Per 0.3 mmol/l	0.93 (0.88–0.98)	0.01
**log_e_CRP**	Per 0.1 log	0.99 (0.97–1.02)	0.87
**eGFR**	Per 15 ml/min/1.73m^2^	1.16 (1.05–1.29)	0.003

* **OR (95% CI)**: odds-ratio (95% confidence interval) of having a glutathione peroxidase activity (GPx-3) activity above the median, estimated using age and gender-adjusted logistic regression. **eGFR**, estimated Glomerular Filtration Rate from MDRD (Modification of the Diet in Renal Disease) formula; **log_e_CRP**, logarithmic of high-sensitivity C-Reactive Protein. HT: hypertensive subjects; NT: normotensive subjects.

## Discussion

The POLA cohort with its extensive clinical phenotyping and elimination of baseline cardiovascular disease, has permitted the examination of the relationship between retinal vascular caliber and biomarkers of oxidative stress and inflammation adjusting for a wide range of cardiovascular risk factors. According to epidemiological studies and meta analyses, larger retinal arterioles indicate a reduced cardiovascular risk while wider venules indicate an increased risk. We demonstrated that retinal arteriolar caliber was related to the activity of plasma GPx-3, a major player in oxidative stress regulation [21], [40], [41], and the association of retinal venular caliber was with inflammation parameters of hsCRP and orosomucoid, independently of other cardiovascular risks factors. These data thus confirm the relationship between a wider retinal venular caliber and inflammation, as well as providing evidence for a novel association between wider retinal arteriolar caliber and oxidative stress quantified by GPx-3 activity. This finding is of particular importance as it suggests that retinal microvasculature, which has been related to carotid arterial stiffness [45] and cardiac remodeling [2], may be particularly sensitive to systemic oxidative stress and systemic inflammation, independently of known cardiovascular risk factors. Thus larger arterial caliber in the retine could be considered as being cardioprotective as it is related to high GPx activity established as being associated with a lower risk of cardiovascular disease.

The present study sheds light on the poorly understood inter-relationships of retinal vascular calibers, cardiovascular risk factors and cardiovascular disease. Smaller retinal arteriolar caliber was related to older age, male gender, lower renal function and hypertensive status. All these associations have already been documented in the literature and the direction of variations in retinal arterial caliber consistently agreed with the established role of the cardiovascular risk factor [46].

Larger retinal arteriolar caliber was associated with current cigarette smoking in the present analysis. Kifley A. et al. [47] also reported both cross-sectional and longitudinal associations between smoking and wider arteriolar caliber. While the precise mechanisms involved in retinal vascular dilation in smokers are as yet unclear, they may relate to reduced oxyhemoglobin and tissue hypoxia, nicotine-induced changes in vessel autoregulation, and secondary polycythemia [48], [49]. Endothelial dysfunction is also a consequence of smoking and could potentially explain our findings [50], [51].

On one hand, larger venules seemed to be associated with higher cardiovascular risk when linked with greater BMI and current cigarette smoking. However retinal venular caliber decreased with older age and poorer renal function. An inverse association between age and retinal vascular calibers has been demonstrated in several population studies [6]–[8]. In the Blue Mountains [6], and the Beaver Dam studies [7] retinal venular caliber decreased by 1.8 to 4.8 µm with each decade of age. Besides, in the present study as well as the ARIC study [52], smaller retinal venular caliber was associated with poorer renal function even after adjustment for age. In a study conducted in healthy kidney donors who underwent a needle biopsy before nephrectomy, the proportion of vessels with arteriosclerosis increased from 45% in young subjects (age 19 to 32 years) to 89% in older subjects (age 65 to 76 years) [53].

We hypothesis that retinal venular caliber decreases with age and thus an aging of intrarenal vasculature. However when exposed to risk factors such as obesity, inflammation and cigarette smoking, venules may undergo a pathologic dilatation that is associated with an increased risk of stroke [14]. These findings should benefit from cellular and molecular biology investigations to improve our knowledge of human microcirculation. The association of GPx-3 activity with arterial vascular caliber was only slightly modified after adjusting for cardiovascular risk factors demonstrating a possible specific effect of the oxidative stress on arterial vascular remodeling. In agreement with clinical findings, where higher GPx activity has been inversely associated with cardiovascular events (both stroke and coronary artery disease) [24]–[28], the present study found higher GPx-3 activity to be cardio-protective, being related to younger age, normotensive status, lower BMI, higher HDL-cholesterol, lower triglycerides, and better renal function.

Oxidative stress is recognized as a key pathogenic process in cardiovascular disease [19], [20]. GPx-3 activity having already been shown to be implicated in arterial thrombosis and stroke in clinical studies [19], [40]. We have furthered understanding of this process by highlighting its influence on early arteriolar changes in subjects showing no clinical signs of atherosclerosis. The present findings are also consistent with recent experimental data on GPx-3 activity. Use of a genetic mouse model to investigate the changes in vessel diameter after methacholine infusion revealed a significant increase in vessel diameter in GPx-3(+/+) mice in contrast with a decrease in GPx-3(–/–) mice [21]. A deficiency in GPx-3 has been associated with an increase in peroxide-related oxidants and decreased available nitric oxide, believed to promote platelet activation [41]. The functional significance of SOD2 vascular activity is not well known [54]. In the present study, SOD2 was not significantly associated with either arterial or venular retinal vascular calibers.

Atherosclerosis is also highly related to inflammatory mechanisms. Our results confirm the findings of previous epidemiological studies which have consistently shown an association between wider retinal venular caliber and elevated levels of systemic inflammatory markers [29]–[31]. Indeed, we observed a significant relationship between venular caliber and the inflammation parameters of hsCRP and orosomucoid. A clear pathophysiological explanation for this association is lacking. It is known that in diabetic retinopathy, the retinal venules become wider [55]. Other examples of venular dilatation are patients with central retinal vein occlusion [56], carotid artery narrowing leading to venous stasis retinopathy, or deep venous thrombosis in the legs, which are more vulnerable to atherosclerosis. It has been suggested that hyperlipidemia, platelet activation, and blood coagulation are involved in the development of both atherosclerosis and venous thrombosis [57]. Whether atherosclerosis induces venular changes, both share common risk factors, or they occur independently of each other still remains to be determined.

The strengths of this study include the precise measurement of retinal vascular caliber, the availability of a cohort free of cardiovascular events and the measurement of a wide range of cardiovascular risk factors as well as biochemical inflammation and oxidative stress parameters. However, several limitations of this study should be noted. The cross-sectional nature of the analyses has precluded observation of the temporal sequence of the reported associations among the cardiovascular risk factors, biochemical parameters and retinal vascular caliber. Further longitudinal analyses are required to assess the determinants of microvascular remodeling as evaluated by retinal vessels.

The advanced age of the population could limit the generalization of the present results to younger persons and studies among middle-aged individuals are required. On the other hand a population of young elderly people is more inclined to experience a cardiovascular event and is therefore the principal target of prevention programmes. Exclusion of subjects with cardiovascular diseases was performed based on medical history. Patients in whom a silent myocardial infarction may have been missed could have led to an over estimation of the present findings. Compared with other population studies of retinal vascular caliber analysis, the POLA study of only 1224 participants may have had lower statistical power thus underestimating associations. However except for diabetic status, the main established associations between retinal vascular calibers and cardio-vascular risk factors were confirmed in this elderly cohort [46]. In the present analysis, although a the high number subjects could not be included in the statistical analysis, it is unlikely that this selection influenced data analyses since no differences were found with included participants, except for age.

In conclusion, data from this elderly cohort indicate that retinal vascular calibers are related to systemic inflammation and a novel parameter, GPx-3 activity. The non-invasive assessment of this biomarker could serve clinically for early diagnosis and thus guide medical decisions in relation to preventive treatment for individuals at risk of cardiovascular disease. Our data also suggest that an assessment of retinal vascular calibers may offer new insights into the pathophysiology of subclinical vascular processes and thus contribute to research and clinical investigations. The natural history of microvascular remodeling and its determinants remain to be investigated in longitudinal and intervention studies.
